# Equine Mx1 Restricts Influenza A Virus Replication by Targeting at Distinct Site of its Nucleoprotein

**DOI:** 10.3390/v11121114

**Published:** 2019-12-02

**Authors:** Urooj Fatima, Zhenyu Zhang, Haili Zhang, Xue-Feng Wang, Ling Xu, Xiaoyu Chu, Shuang Ji, Xiaojun Wang

**Affiliations:** 1 State Key Laboratory of Veterinary Biotechnology, Harbin Veterinary Research Institute, Chinese Academy of Agricultural Sciences, Harbin 150069, China; uroojfatima.hvri@yahoo.com (U.F.); zhangzhenyu@caas.cn (Z.Z.); zhanghaili@caas.cn (H.Z.); wangxuefeng@caas.cn (X.-F.W.); 18266423976@163.com (L.X.); chuxiaoyuer@foxmail.com (X.C.); jishuang9096@163.com (S.J.); 2 Advanced Institute for Medical Sciences, Dalian Medical University, Dalian 116044, China

**Keywords:** MxA, equine Mx1, influenza A viruses, polymerase activity, interspecies transmission, nucleoprotein, equine influenza

## Abstract

Interferon-mediated host factors myxovirus (Mx) proteins are key features in regulating influenza A virus (IAV) infections. Viral polymerases are essential for viral replication. The Mx1 protein has been known to interact with viral nucleoprotein (NP) and PB2, resulting in the influence of polymerase activity and providing interspecies restriction. The equine influenza virus has evolved as an independent lineage to influenza viruses from other species. We estimated the differences in antiviral activities between human MxA (huMxA) and equine Mx1 (eqMx1) against a broad range of IAV strains. We found that huMxA has antiviral potential against IAV strains from non-human species, whereas eqMx1 could only inhibit the polymerase activity of non-equine species. Here, we demonstrated that NP is the main target of eqMx1. Subsequently, we found adaptive mutations in the NP of strains A/equine/Jilin/1/1989 (H3N8_JL89_) and A/chicken/Zhejiang/DTID-ZJU01/2013 (H7N9_ZJ13_) that confer eqMx1 resistance and sensitivity respectively. A substantial reduction in Mx1 resistance was observed for the two mutations G34S and H52N in H3N8_JL89_ NP. Thus, eqMx1 is an important dynamic force in IAV nucleoprotein evolution. We, therefore, suggest that the amino acids responsible for Mx1 resistance should be regarded as a robust indicator for the pandemic potential of lately evolving IAVs.

## 1. Introduction

Influenza A viruses (IAVs), commonly termed flu viruses, belong to the *Orthmyxoviridae* family and are broadly associated with acute febrile respiratory disease in many animals. Wild aquatic birds are believed to be the reservoir for these viruses [1,2]. However, although IAVs were initially limited to wild waterfowl, these viruses crossed the species barrier and have been spreading to other populations, including mammalian species, and have established independent lineages [1,3,4].

IAV possess a segmented and negative sense single-stranded RNA genome. The eight genomic segments of IAV encode at least 10 viral proteins. IAV derives its envelope from the host cell plasma membrane and contains three transmembrane proteins: Two surface glycoproteins termed as hemagglutinin (HA) and neuraminidase (NA), along with matrix protein 2 (M2). Two non-structural proteins NS1 and NS2, collectively termed as nuclear export protein (NEP) are associated with matrix protein 1 (M1) and viral ribonucleoprotein complexes (vRNPs). The eight vRNPs comprise of eight negative-strand RNA segments associated with the nucleoprotein (NP) and three RNA-dependent RNA polymerase (RdRp) subunits (PA, PB1, and PB2) [5]. Viral vRNPs are considered as minimal functional units required for early transcription (viral mRNA synthesis) and viral replication (vRNA synthesis) [6].

Myxovirus resistance proteins, including MxA and MxB in humans (named Mx1 and Mx2 in other animals), belong to dynamin-like GTPases family and are considered to be cell-autonomous host restriction factors of the innate immune system against many viral pathogens. Both the type-I and type-III interferons stimulate MxA and MxB expression during innate immune signaling [7,8]. The MxA protein is an indicator gene that is induced following an interferon action and halts a broad range of viral pathogens including DNA and RNA viruses (mainly *Orthomyxoviruses* such as influenza [9,10], measles [11], La Crosse [12], and *Hantaan* viruses [13]). HuMxA is a potent interspecies barrier for influenza viruses from other species [8,10,14].

MxA is a dynamin-like large GTPase comprised of an N-terminal globular GTPase domain, a bundle signaling element (BSE), and a C-terminal helical stalk. MxA can form stable tetramers and oligomers, which assemble in a criss-cross manner via the stalk [15,16]. Although the exact mechanism of Mx1-mediated immunity to influenza viruses is still unclear, a proposed possible mechanism suggests that, upon entry of viral infectious particles, MxA recognizes the incoming vRNPs and starts to self-assemble into rings, resulting in a higher-order oligomeric complex that blocks the vRNP function [16,17]. Viral NP is known to be a target of human and mouse MxA whereas PB2, which is associated with NP in the viral nucleocapsid, may serve as an additional target [6,18,19].

MxA represents a considerable barrier against the zoonotic introduction of avian influenza viruses into the human population [7]. Interestingly, several investigations reported the antiviral properties of MxA from a human and mouse [10,20,21], porcine [22,23], and bovine [14,24,25,26] origin. The interspecies transmission would have occurred when avian-origin IAV acquired certain mutations in the NP which overcome the MxA restriction [20,22,27]. A few of these mutations were already found in circulating IAV strains before they were even reported to cross the species barrier [20,22], but in many cases, the adaptational mutations occurred subsequent to infection and transmission. Indeed, recent studies indicated that IAVs which successfully established stable lineages in humans acquired adaptive mutations in the NP [20,28]. It was reported that a mutated H7N7 IAV carrying human signature NP mutations was more virulent in transgenic mice than the parental virus [29].

The equine influenza virus underwent an independent evolution pattern that can be compared with viruses from other species [30]. Equine IAVs can be classified into two main subtypes: H7N7 (a prototype virus A/equine/H7N7/Prague/56 that was isolated in 1956) [31] and H3N8 (a prototype virus A/equine/H3N8/Miami/63 that was isolated in 1963). Equine H7N7 viruses have not been isolated since 1990 and it is believed that they are no longer circulating in the equine population. Very little genetic exchange between the equine H3N8 virus subtype and viruses from other host species has been reported [32], which was thought to signify that horses were an endpoint until the H3N8 virus was acknowledged as being of canine and equine origin. Whether the equine Mx1 is functional and plays any role in interspecies restriction remains largely known. In this study, we investigated the interspecies restriction of eqMx1 to IAVs from different species. We found that different IAV strains were Mx1 or MxA sensitive to varying degrees. We further identified that 1) viral NP interacts with eqMx1 and determines the sensitivity to Mx1, and 2) single amino acid substitution at site 52 of the NP has a key role in determining this interaction and binding to Mx1. Our results suggest that the IAVs in the equine population have gained important mutations in the NP which enable a higher chance of eqMx1 resistance.

## 2. Materials and Methods

### 2.1. Cells and Viruses

Human embryonic kidney 293T (HEK293T) and Madin–Darby canine kidney (MDCK) cells were maintained in Dulbecco′s Modified Eagle′s Medium (DMEM) (Sigma Aldrich, St. Louis, MO, USA) supplemented with 10% foetal bovine serum (FBS, Wisent, Canada) and 1% antibiotics (100 units/mL of penicillin and 100 µg/mL of streptomycin; ThermoFisher, Waltham, MA, USA). The equine monocyte-derived macrophages (eMDMs) were prepared from equine peripheral blood mononuclear cells (PBMCs) as described previously [33]. Briefly, three healthy equids (horses) were used to collect the blood. The buffy coat was separated from blood samples by centrifugation at 1000 rpm for 10 min. Later, the buffy coat was used to isolate the PBMCs by centrifugation using a HybriMax Histopaque cushion (Sigma Aldrich, St. Louis, MO, USA) (d = 1.077 g/cm^3^). The collected PBMCs were washed three times with PBS, resuspended, and maintained in RPMI 1640 (HyClone, Logan, Utah, USA) supplemented with 30% horse serum and 30% fetal bovine serum (FBS) (HyClone, Logan, Utah, USA). Aliquots of these isolated PBMCs were later seeded into tissue culture flasks at a density of 5 × 10^6^ cells/cm^2^ and were further incubated in a humidified chamber at 37 °C with 5% CO_2_. At 24 hr post-incubation, the non-adherent cells which were found floating in the medium were discarded and the adherent monocytes were again incubated for the next 3 days in order to allow their differentiation into eMDMs. The cell culture atmosphere was maintained at 37 °C with 5% CO_2_.

Wild type Influenza A/equine/Jilin/1/89 (H3N8_JL89_) and recombinant viruses were generated using a plasmid-based reverse genetics system as previously described [34,35]. The generated wild-type virus and JL89-H52N-NP mutant virus were further propagated in 9-day-old embryonated chicken eggs at 35 °C for 72 hr, and allantoic fluid was harvested and centrifuged, and a hemagglutination test was performed to confirm the presence of the virus. Viruses from positive-testing samples were filtered through a 0.2 µm syringe filter (Millex, Merck, Ireland), and the viral titers were determined using the TCID_50_ method of Reed and Muench [36]. Viruses were subsequently stored in small aliquots at −80 °C until further use.

### 2.2. Plasmids and Antibodies

In order to clone the cDNA derived from the total RNA of eMDMs, we used reverse-transcription PCR (RT-PCR) to amplify eqMx1. The eMDMs were treated with equine IFN-α1 (100 ng/μL) (Kingfisher Biotech, Minnesota, USA) for 24 hr. The primer sets were constructed following the genome sequences of *Equus caballus* myxovirus (Mx) dynamin-like GTPase 1 (Mx1), transcript variant X1, gene (GenBank accession no. XM_005606071.3), and the sequence of primers can be found in supplementary data (Table S1). Then, the fragments were successfully cloned in pcDNA3.1-HA vector (pcDNA3.1 (+) vector (Invitrogen, ThermoFisher, Waltham, MA, USA) that possessed 2X HA tags situated on the C-terminus and also p3X-Flag-CMV vector (Sigma Aldrich, St. Louis, MO, USA) that contained 3X Flag tags located at the N-terminus. The huMxA gene was acquired from Summus Co. (China) and was also successfully cloned using pcDNA3.1 (+).

The regents used in this study were kindly provided by the following personals: H1N1 human influenza virus A/WSN/1933 (WSN) by Dr. Kawoaka, and plasmids of H7N9 A/chicken/Zhejiang/DTID-ZJU01/2013 (H7N9_ZJ13_) and H1N1 human IAV A/Sichuan/01/2009 (H1N1_SC09_) by Dr. Hualan Chen. The equine influenza viruses H3N8_XJ07_ (A/equine/Xinjiang/1/2007) and H3N8_JL89_ were acquired from the previously preserved viral stock in our lab.

Site-directed mutants of NP sequences were generated using overlapping PCR and identified by DNA sequencing. Mutants of pcAGGS-H7N9-NP and pEF-JL89-NP were constructed according to the online In-Fusion^®^ HD Cloning Kit (Clontech, Felicia, CA, USA) user manual (http://www.clontech.com/CN/Products/Cloning_and_Competent_Cells/Clonin_Kits/xxclt_searchResults.jsp). Briefly, the fragments of the pCAGGS/pcDNA3.1 vector and each target gene were amplified and were then fused using the In-Fusion^®^ HD Enzyme kit (Clontech, Felicia, CA, USA). To create the N-H7N9xJL89-C plasmid, pcAGGS-H7N9-NP was used as the template to amplify the pCAGGS vector along with the N-terminal (1–200) of H7N9. This sequence was then fused with the JL89-NP C-terminal (201–480) fragment, where pEF-JL89-NP was used to amplify the inserted C-terminal (201–480) fragments. To obtain N-JL89xH7N9-C plasmids, pEF-JL89-NP was used as the template to amplify the pEF-JL89 vector backbone sequence along with the N-terminal (1–200) of JL89 and the inserted C-terminal (201–480) fragment of H7N9 was amplified from the pCAGGS-H7N9-NP sequence. The amplified vector and insert fragments were then fused together as described earlier. Single point mutations were also created by overlapping PCR in the same way. The constructed plasmids were transformed into DH5-alpha or Stable II competent cells according to the manufacturer’s protocol (ThermoFisher, Waltham, MA, USA). Once the transformation was successful, the plasmids were extracted using PureLink HiPure plasmids DNA purification kits (Invitrogen, ThermoFisher, Waltham, MA, USA). All generated plasmids were confirmed by PCR and sequencing. The sequence of all the primers is available in supplementary data (Tables S2 and S3).

### 2.3. Polymerase Reconstitution Assay/Minireplicon Assay

In order to determine polymerase activity, we transfected the minigenome reporter (enclosing a firefly luciferase gene linked with non-coding regions of the HA gene of influenza containing human polI as the promoter and a mice terminator sequence) with NP and viral polymerase expression plasmids. We generated the chimeric mutants of the NP gene by overlapping PCR and verified it by the sequencing technique. To predict the viral polymerase activity due to mutations in the NP, a previously described protocol was followed [37]. Briefly, HEK293T cells were seeded in 24-well plates and co-transfected with expression plasmids of all viral polymerases i.e., PB1 (40 ng), PB2 (40 ng), PA (20 ng), and NP (80 ng), together with 40 ng of minigenome reporter (FF-Luc) and 10 ng of *Renilla* luciferase expression plasmids (pRL-TK, as an internal control) using polyjet transfection reagent (Invitrogen, ThermoFisher, Waltham, MA, USA) according to the manufacturers’ protocol. Cells were maintained at 37 °C. To perform the cell lysis, we employed 200 μL of 1X reporter lysis buffer (Promega, Madison, USA) after 24 hr of transfection. To measure the luciferase activities of *Renilla* and Firefly, we employed the commercially available Dual-luciferase kit (Promega, Madison, USA) using Centro XS LB 960 luminometer (Berthold Technologies, Oak Ridge, Tennessee, USA). The levels of polymerase protein expression were detected using western blotting, with specific mouse monoclonal antibodies for NP and anti-HA tag antibody (Sigma Aldrich, St. Louis, MO, USA) for eqMx1-HA and huMxA-HA proteins. In the present study, all experimentation was performed independently at least thrice. The calculated results demonstrate mean ± SEM within one experiment.

### 2.4. Co-Immunoprecipitation Assay

Cells were incubated in a T-75 cell culture flask (ThermoFisher, Waltham, MA, USA) until a confluency of 70% was attained. HEK293T cells were then co-transfected with p3XFLAG-tagged eqMx1 (4 μg) together with PB1, PB2 (2 μg each), PA, FF-Luc (1 μg each), and NP expression plasmids either from (1) H7N9, and its mutants H7N9-S34G and N52H; or (2) H3N8_JL89_ and its mutants JL89-G34S and H52N; or (3) an empty control vector (4 μg each) by using PolyJet™ In Vitro DNA transfection reagent (SignaGen, Rockville, MD, USA) according to the manufacturer’s instructions. At24 hr post-transfection, co-immunoprecipitation was performed as previously described [38]. Briefly, transfected cells were lysed using an ice-cold lysis buffer (50 mM of Tris-HCL [pH 8], 150 mM of NaCl, 5 Mm of EDTA, and 1% NP-40), protease inhibitors (1:100), and freshly prepared 2 M N-ethylmaleimide (NEM) at 1:80 (Sigma Aldrich, St. Louis, MO, USA) and centrifuged at 4 °C for 10 min at 13,000× *g*. Post centrifugation, using Anti-FLAG M2 magnetic beads (Sigma Aldrich, St. Louis, MO, USA, M8823) or Anti-NP magnetic beads (made by incubation of MCE protein A/G magnetic beads along with the NP antibody from our lab) the lysates were co-incubated for 2 hr at 4 °C. Using a magnetic separator, the resins were harvested and washed thrice with cold PBS. In order to elute the resin-bound materials, we added a 3X Flag peptide and incubated the samples at 4 °C for 30 min, and then the eluted samples were boiled and SDS-PAGE was performed. The samples were shifted on the nitrocellulose membrane. 5% skim milk in Tris-buffered saline (TBS) was used as a blocking buffer for 2 hr and then the membranes were incubated with specific primary antibodies-TBST (TBS with 0.05% Tween20) at room temperature for 2 hr. Washing was performed thrice and the membranes were then incubated in TBST, containing secondary antibody (Sigma Aldrich, St. Louis, MO, USA, 1:10,000) at room temperature for 1 hr. Subsequently, membranes were washed three times in PBST and scanned using a LI-COR Odyssey Imaging System (LI-COR, Lincoln, NE, USA).

### 2.5. Virus Growth Kinetics

The cells were seeded in 6-well plates and the next day, cells were transfected with pcDNA-3.1 eqMx1. 24 hr post-transfection, an infection experiment was performed as previously described [39]. Briefly, after 24 hr, the culture medium was removed and the cells were gently washed twice with preheated phosphate-buffered saline (PBS) and then infected for 1 hr at 37 °C with wild-type (H3N8_JL89_) and/or mutant viruses (JL89-H52N-NP) at a multiplicity of infection (MOI) of 0.001. Viral dilutions were made in DMEM, supplemented with 1 μg/mL of TPCK added trypsin (Sigma Aldrich, St. Louis, MO, USA) and 0.25% BSA (Roche, Basel, Switzerland). Plates were gently shaken every 15 min so that the virus remained equally distributed within the cells. Later, excess virus was removed and cells were gently washed three times with preheated PBS and fresh infection media was added. Finally, the supernatants were collected at 0, 12, 24, 36, and 48 hr post-infection and debris was removed by centrifugation at 1000× *g* for 5 min. The amount of virus in each supernatant was determined either by plaque assay or by determining tissue culture infectivity dose and is expressed here either as PFU per mL or TCID_50_/mL respectively.

### 2.6. Measurement of Gene Expression Using RT-qPCR

Total RNA from the collected supernatants was extracted using the RNeasy plus Minikit (Qiagen, Venlo, Netherland) and were subjected to a one-step real-time quantitative PCR (RT-qPCR) analysis using the AgPath-ID™ One-Step RT-PCR reagents (ThermoFisher, Waltham, MA, USA) according to the manufacturer’s protocol. FAM-labeled probes (EIV-Tq-P) 5′-TCAGGCCCCCTCAAAGCCGA-TAMRA and specific primers targeting the M-gene of IAV, 5′-AGATGAGYCTTCTAACCGAGGTCG-3′ (EIV-Tq-forward) and 5′-TGCAAANACATCYTCAAGTCTCTG-3′ (EIV-Tq-reverse) were used for the amplification of RNA, and relative mRNA expression levels were determined using double-standard curve methods.

### 2.7. Immunofluorescence Assay (IFA)

Briefly, MDCK cells were infected with wild-type (H3N8_JL89_) and/or mutant viruses (JL89-H52N-NP) at an MOI of 0.001 and collected supernatants were used to infect MDCK cells in 96-well plates at various dilutions for 48 hr. Cells were fixed with chilled absolute ethanol for 30 min. The fixed cells were incubated with the anti-NP antibody (from our lab) for 2 hr at 37 °C in a humidified chamber, followed by three washes with phosphate-buffered saline (PBS), and then incubated with fluorescein isothiocyanate- (FITC) labeled goat anti-mouse IgG (Sigma Aldrich, St. Louis, MO, USA) for 1 hr 37 °C. Following three washes with PBS, the cells were examined under an inverted fluorescence microscope (Nikon TE200, Tokyo, Japan).

### 2.8. Evolutionary Analysis

In order to determine the evolutionary relationships between NPs from IAV strains from different hosts, a total of 153 representative nucleotide sequences of viral nucleoproteins from IAVs with different host specificities (including avian, porcine, canine, equine, and human) were retrieved from the GenBank and aligned manually using Bioedit alignment tools. The evolutionary history was inferred by the maximum likelihood method based on the JTT matrix-based model.

### 2.9. Statistical Analysis

In order to perform the statistical analyses, we used GraphPad Prism, version 5 (https://www.graphpad.com/scientific-software/prism/) (San Diego, California, USA). Employing the statistical approaches of one-way ANOVA and Dunnett’s post-test, the statistical differences were analyzed. All experiments were thrice conducted independently. The error bars present standard deviation (SD) or standard error of the mean (SEM) in each experimental group that is documented in the figure legends. The abbreviations are designated as NS not significant, *p* > 0.05, * 0.01 ≤ *p* < 0.05, ** 0.001 ≤ *p* <0.01, *** 0.0001 ≤ *p* < 0.001, and **** *p* < 0.0001 respectively.

## 3. Results

### 3.1. Identification of Cross-Species Antiviral Potential of Human and Equine Mx1

**Figure 1:**
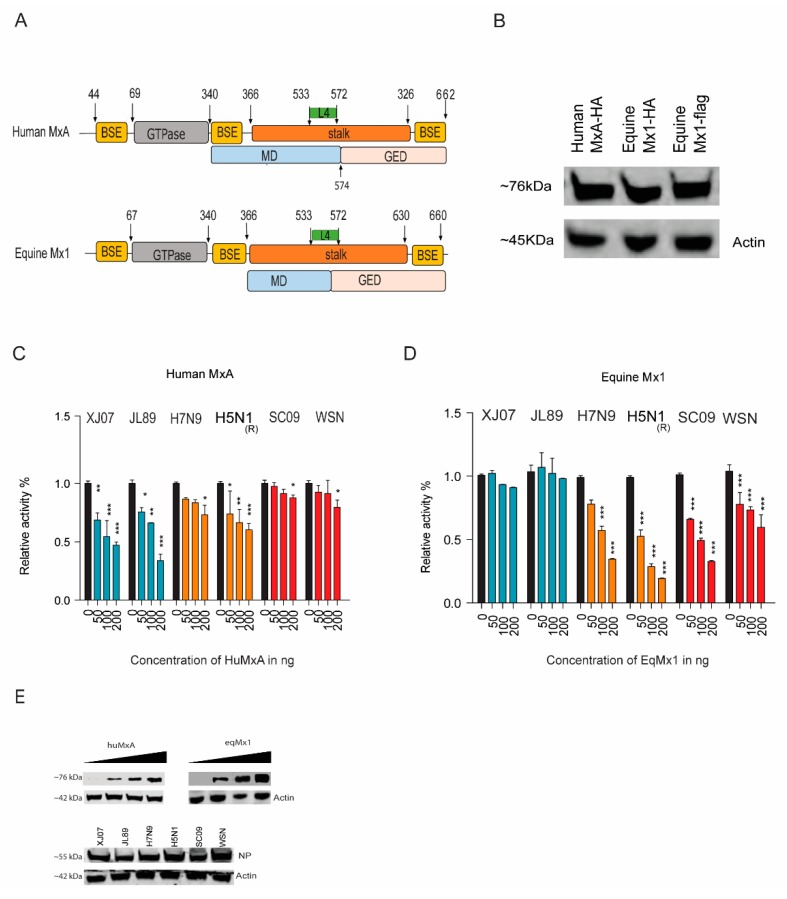
Antiviral activities of human MxA (huMxA) and equine Mx1 (eqMx1). (**A**) Structure-based domain representation of human MxA and equine Mx1. The GTPase is shown in grey, the middle domain (MD) is blue, and the GTPase effector domain (GED) is shown in pink. The GTPase domain is connected to the MD and GED through bundle signaling element (BSE) (yellow) and the stalk is colored orange. The arrows in the schematic denote the first and last visible residues in the structure. (**B**) Expression of plasmids by western blotting. The expression of huMxA-pcDNA3.1-HA, eqMx1-pcDNA3.1-HA, and p3X-FLAG-eqMx1 plasmids. The figures represent the bands of expressed proteins as visualized using western blotting, with antibodies used against the hemagglutinin (HA) tag and flag tag. (**C**,**D**) Relative luciferase activities of different influenza A virus (IAV) strains. Human embryonic kidney 293T (HEK293T) cells were co-transfected with expression plasmids of PB1 (40 ng), PB2 (40 ng), PA (20 ng), and nucleoprotein (NP) (80 ng) from different IAV strains, together with 40 ng of minigenome reporter (FF-luc) and 10 ng of *Renilla* luciferase expression plasmids (pRL-TK, as an internal control) in the presence of HA-tagged pcDNA-3.1 eq-mx1 (**C**) or pcDNA-3.1 huMxA (**D**) and an empty control vector at increasing concentrations of 0, 50, 100, and 200 ng each. After 24 hr of transfection, cells were lysed using a 1X reporter lysis buffer. Firefly and *Renilla* luciferase activities were measured. The resulting relative activity in the presence of either H7N9_ZJ13_ or H3N8_JL89_ was set to 100%. (**E**) The western blot analysis was performed to determine the expression levels of eqMx1 or huMxA and NP expression plasmids. (Statistical differences between samples are indicated, according to a one-way ANOVA followed by a Dunnett’s test; NS = not significant, * 0.01 ≤ *p* < 0.05, ** 0.001 ≤ *p* < 0.01, *** *p* < 0.001. Error bars represent the SEM within one representative experiment).

MxA (or Mx1) as one of the major interferon-stimulated genes (ISGs) is responsible for counteracting many invading viruses, especially those belonging to *Orthomyxoviridae*. To identify the eqMx1′s function in the restriction of IAVs, we first cloned the equine Mx1 from eMDMs. By using bioinformatics prediction software (https://www.uniprot.org, SWISS-MODEL, and Phyre2), eqMx1 was predicted to be structurally similar to huMxA [15] (Figure 1A). The eMDMs were treated with equine IFN-α1 (100 ng/μL) (Kingfisher Biotech, Minnesota, USA) for 24 hr to enhance the basic expressing level of eqMx1, and a 1983-bp fragment was amplified and cloned into a pcDNA3.1-HA vector [pcDNA3.1 (+)] (Invitrogen, ThermoFisher, Waltham, MA, USA) with 2X HA tags at the C-terminal. The expressions of eqMx1 and huMxA were verified by western blotting (Figure 1B). Polymerase assay was then used to evaluate Mx1′s effect on the replications of IAV strains from different host species, including human IAVs H1N1 (A/Sichuan/01/2009 and A/WSN/1933), avian IAVs H7N9 (A/chicken/Zhejiang/DTID-ZJU01/2013) and H5N1 (A/chicken/Scotland/1959), and equine IAVs H3N8 (A/equine/Xinjiang/1/2007 and A/equine/Jilin/1/1989). Mx1 or MxA resistance is defined here as the comparative activity of the viral polymerase in the presence of Mx1 divided by the activity calculated in the absence of Mx1. The results suggest that huMxA has antiviral potential against the IAV strains from non-human species i.e., avian and equine strains, whereas eqMx1 could only restrict the polymerase activity of non-equine species i.e., avian and human. The antiviral activity of eqMx1 to avian IAVs (H7N9_ZJ13_ and H5N1_(R)_) was slightly stronger than that of huMxA (Figure 1C,D). The expression of all the plasmids was determined with western blotting (Figure 1E). The results suggest that huMxA has antiviral potential against the IAV strains of avian and equine but not a human origin, whereas eqMx1 could inhibit the polymerase activity of avian and human but not equine stains.

### 3.2. The Viral Nucleoprotein as a Possible Target of Mx1 Action

**Figure 2:**
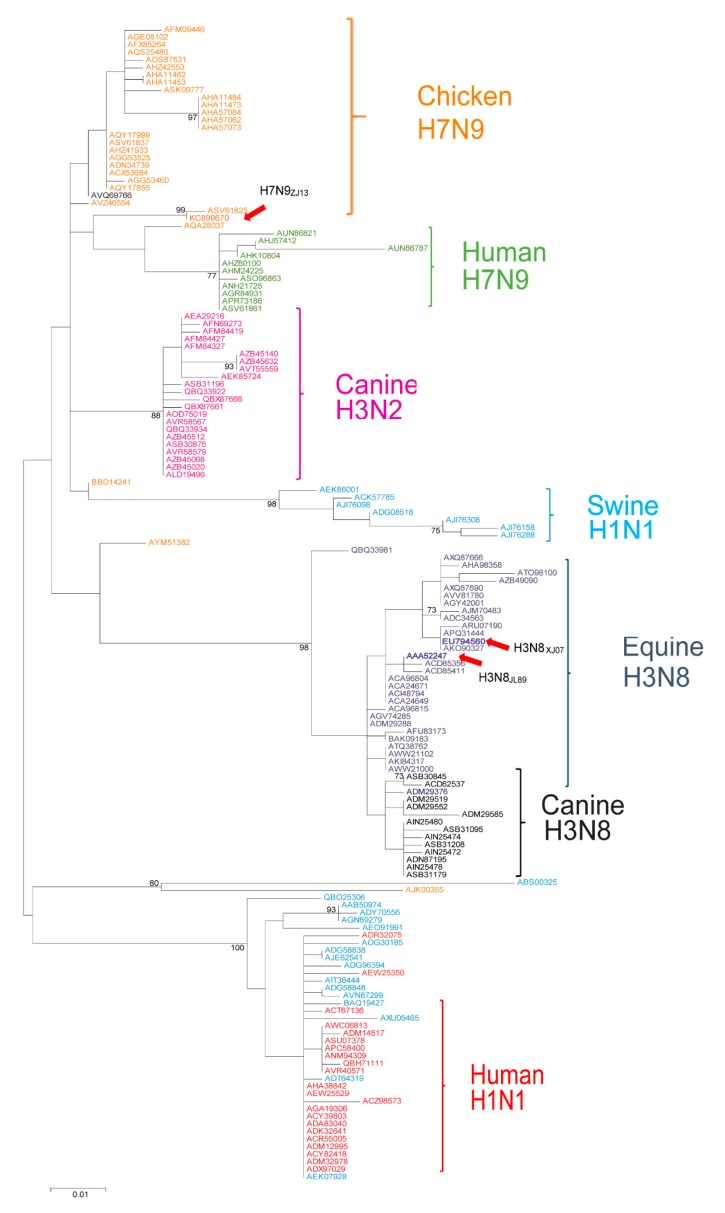
Phylogenetic analysis demonstrates the relationship of influenza A virus NP from distinct hosts. Molecular phylogenetic analysis of evolutionary relationships among different viral strains was inferred using the maximum likelihood strategy. The analysis was conducted using the JTT matrix-based model. The tree with the highest log likelihood (2519.6632) is shown. The percentage of trees in which the associated taxa clustered together is shown next to the branches. The initial tree(s) for the heuristic search was obtained automatically by applying neighborhood Join and BioNJ algorithms to a matrix of pairwise distances estimated using the JTT model and then selecting the topology with the superior log-likelihood value. A discrete Gamma distribution was used to model the evolutionary rate differences among sites [five categories (+G, parameter = 0.4056)]. The tree is drawn to scale, with branch lengths representing the number of substitutions per site. The analysis involved 153 sequences. All positions containing gaps and missing data were eliminated. The final dataset comprised a total of 304 positions. Evolutionary analyses were conducted in MEGA7 software [40].

Viral NP is a major target of MxA and the adaptive mutations of NP enable the virus to escape from MxA restriction [20,21,41]. Analysis of a total of 153 representative nucleotide sequences of viral NPs from IAVs with different host specificities (including avian, swine, canine, equine, and human) showed that the NP proteins generate diverse lineages in a host-species-specific manner (Figure 2). Considering the diversity of Mx1 proteins and the different activities on viruses from different hosts (Figure 1), we hypothesized that the NP from equine viruses may have evolved a specific signature of adaptation to counter eqMx1, and this “signature” of the equine virus may not be able to overcome the restriction of human MxA.

**Figure 3:**
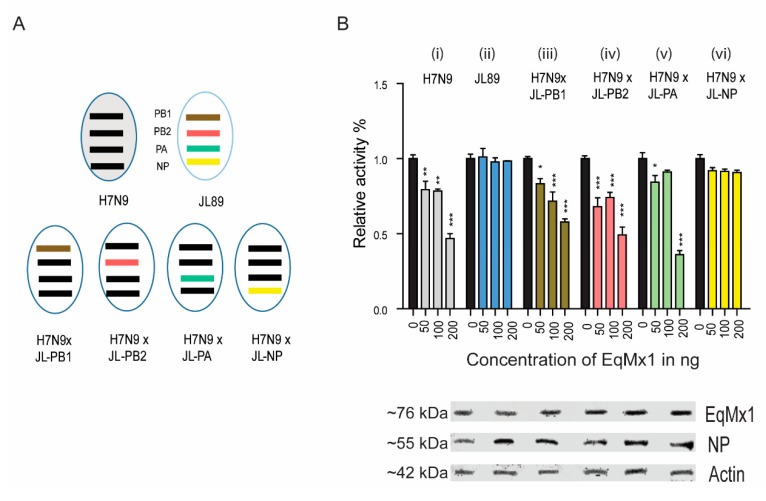
The viral NP is responsible for the sensitivity to eqMx1. (**A**) Schematic representation of the assortment of viral polymerases. The four polymerases (PB1, PB2, PA, and NP) from chicken H7N9_ZJ13_ were swapped one by one with the equivalent polymerases from A/equine/Jilin/1/1989 (H3N8_JL89_), and each group of assorted plasmids was co-transfected into HEK293T. (**B**) Relative Luciferase activities of combination sets of IAV against eqMx1. Cells were co-transfected with expression plasmids of the polymerase PB1 (40 ng), PB2 (40 ng), PA (20 ng), and NP (80 ng) in six different groups, (i) all polymerases of H7N9_ZJ13_, (ii) all polymerases of H3N8_JL89_, (iii) PB2, PA, NP of H7N9_ZJ13_ and PB1 of H3N8_JL89_, (iv) PB1, PA, NP of H7N9_ZJ13_ and PB2 of H3N8_JL89_, (v) PB1, PB2, NP of H7N9_ZJ13_ and PA of H3N8_JL89_, (vi) PB1, PB2, PA of H7N9_ZJ13_ and NP of H3N8_JL89_, together with 40 ng of minigenome reporter (FF-luc) and 10 ng of *Renilla* luciferase expression plasmids (pRL-TK, as an internal control) in the presence of HA-tagged pcDNA-3.1 eqMx1 or an empty control vector at an increasing concentration 0, 50, 100, and 200 ng each. After 24 hr of transfection, cells were lysed using a 1X reporter lysis buffer. Firefly and *Renilla* luciferase activities were measured. The resulting relative activity in the presence of either H7N9_ZJ13_ or H3N8_JL89_ was set to 100%. The western blot analysis shown in (**B**) was performed to determine the expression levels of eqMx1 and NP. (Statistical differences between samples are indicated, according to a one-way ANOVA followed by a Dunnett’s test; NS = not significant, * 0.01 ≤ *p* < 0.05, ** 0.001 ≤ *p* < 0.01, *** *p* < 0.001. Error bars represent the SEM within one representative experiment).

To confirm this idea, we used a mini-replicon system-based approach to identify the target sequence of NP for eqMx1. We swapped expression plasmids between the different strains H7N9_ZJ13_ and H3N8_JL89_ to determine whether an Mx1-sensitive mini-replicon system could be converted into a more resistant system and vice versa (Figure 3A). The replacement PB1, PB2, or PA from H3N8_JL89_ into H7N9_ZJ13_, did not change the polymerase activity of the re-assorted complex to a large extent. However, the replacement by H3N8_JL89_ NP resulted in a complete reversal of the polymerase activity of H7N9_ZJ13_ equivalent to the actual H3N8_JL89_ polymerase system (Figure 3B). Thus, our approach identified the IAV NP as a possible target of eqMx1.

### 3.3. Identification of Residues in NP of the H3N8_JL89_ Influenza A Virus Responsible for Resistance to eqMx1

**Figure 4:**
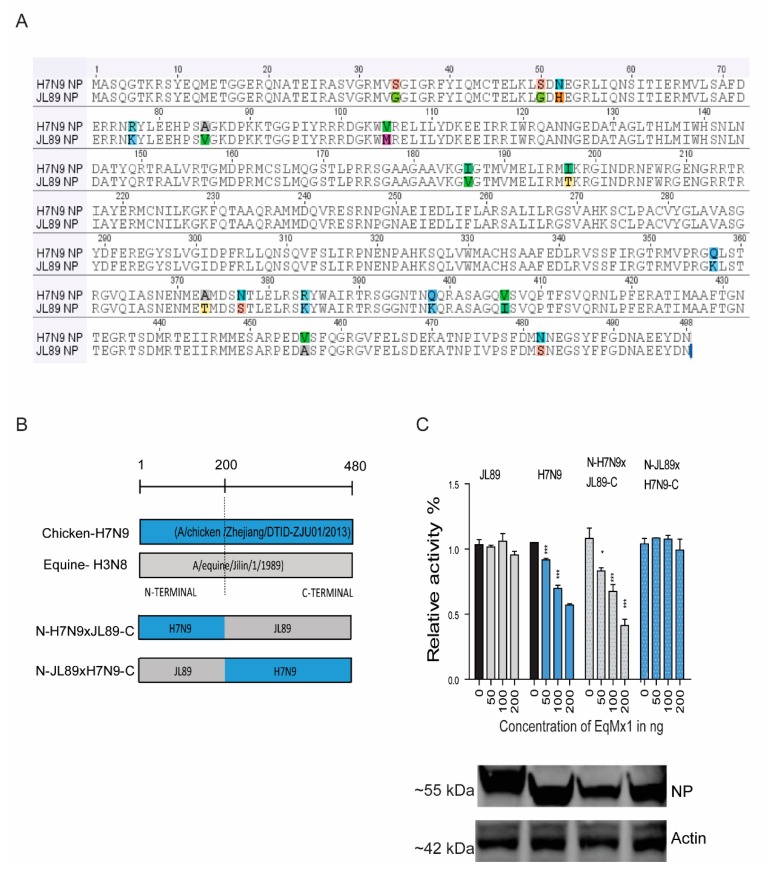
The N terminal of the NP determines the resistance to the restriction of eqMx1. (**A**) The sequence alignment of NP from H7N9_ZJ13_ and H3N8_JL89_ showed the differences in AAs at 16 different positions. (**B**) The schematic presentation of mutant NP sequences generated using overlapping PCR. The NP sequences of H3N8_JL89_ and H7N9_ZJ13_ were divided into two segments at the N and C terminals (AAs position N = 1–200; C = 201–480). The figure shows the nucleotide positions where the site-directed mutants of NP sequences were generated. (**C**) The relative luciferase activity of chimeric clones of NP against eqMx1. Constructed site-directed mutants of the NP sequences were tested for their polymerase activities in the presence of an HA-tagged pcDNA-3.1 eqMx1 or pcDNA-3.1-HA empty control vector, transfection complexes of viral polymerases and reporter plasmids were made and the test was performed as described earlier. The expression levels of eqMx1 and NP proteins were assessed using western blotting. (Statistical differences between samples are indicated, according to a one-way ANOVA followed by a Dunnett’s test; NS = not significant, * 0.01 ≤ *p* < 0.05, *** *p* < 0.001. Error bars represent the SEM within one representative experiment).

Next, we evaluated the key residues of NP from the H3N8_JL89_ IAV that confer eqMx1 resistance in the context of the H7N9_ZJ13_ polymerase. The sequence analysis comparing the NPs of H7N9_ZJ13_ and H3N8_JL89_ of avian origin showed 16 amino acids (AAs) variations between the two strains (Figure 4A). Based on the sequence alignment obtained in Figure 4A, two different chimeras were constructed by fusing two NP proteins from H3N8_JL89_ and H7N9_ZJ13_ (Figure 4B) and tested their polymerase activity against eqMx1. An artificial chimera N-JL89xH7N9-C comprised of the N-terminal 200 AAs of the H3N8_JL89_ NP and the C-terminal domain (AAs position 201 to 480) of the H7N9_ZJ13_ NP, behaved like the full-length H3N8_JL89_ NP, indicating that the eight different AAs at the C-terminal of the NP protein did not contribute to the Mx1 resistance phenotype (Figure 4C). However, a chimeric construct N-H7N9xJL89-C with the replacement of the N-terminal of H3N8_JL89_-NP by 200 AAs from H7N9_ZJ13_ made it more sensitive towards inhibition by eqMx1 in contrast to the actual H3N8_JL89_-NP. Conversely, the opposite mutant N-JL89xH7N9-C that had an insertion of 200 AAs from H3N8_JL89_ into the N-terminal of H7N9_ZJ13_-NP lost its activity as compared to the wild type H7N9_ZJ13_-NP, indicating that the N-terminal played a key role in the determination of resistance or sensitivity towards eqMx1 (Figure 4C).

**Figure 5:**
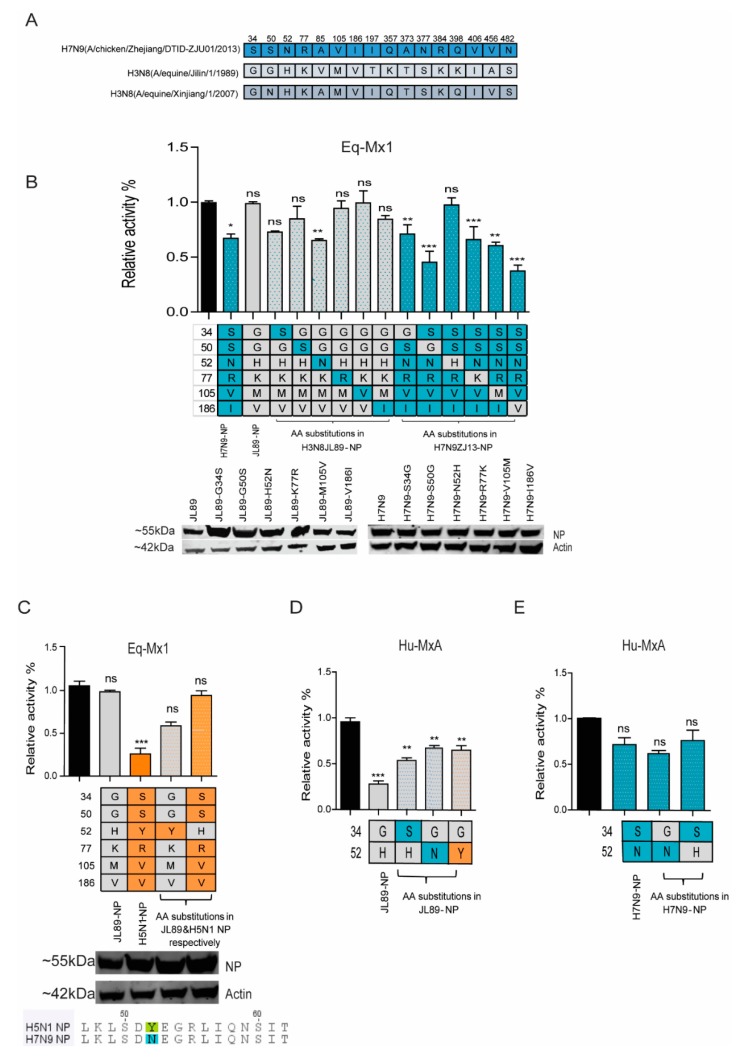
Single amino acid mutation of the NP confers resistance to eqMx1. (**A**) The different AAs in the NP of H7N9_ZJ13_, H3N8_JL89_, and H3N8_XJ07_. (**B**–**E**) HEK 293T cells were transfected with a firefly minigenome reporter, *Renilla* expression control, and respectively indicated point mutants of the NP from either H3N8_JL89_ polymerase (**B**) or H7N9_ZJ13_ polymerase (**B**) or H5N1 polymerase (**C**) in the presence of eqMx1 (0, 200 ng). Polymerase activity of mutants with point mutations at sites 34 and 52 of the NP from both H3N8_JL89_ (**D**) and H7N9_ZJ13_ (**E**) was measured against huMxA (0, 200 ng). Luciferase activity was measured at 24 hr post-transfection. The polymerase activity observed in the presence of the HA-tagged eqMx1 was normalized to an empty control vector (black bar). The resulting relative activity in the presence of either H7N9_ZJ13_ (blue) or H3N8_JL89_ (grey) was set to 100%. The sequence analysis of the aforementioned IAV strains is shown in C. (**A**–**E**) data are firefly Luciferase gene activity normalized to that of *Renilla*. (Statistical differences between cells are indicated, following a one-way ANOVA and subsequent Dunnett’s test; NS = not significant, * 0.01 ≤ *p* < 0.05, ** 0.01 ≤ *p* < 0.01, *** *p* < 0.001. Error bars represent the SEM of the replicates within one representative experiment). The expression levels of all NP point mutant proteins were assessed by western blotting.

Out of eight substitutions of NP, two positions V85A and T197I were not selected for mutations as when the sequence of two strains of H3N8, H3N8_JL89_, and H3N8_XJ07_, were compared, the H3N8_XJ07_ (GenBank accession no. EU794560.1) contained the same amino acids at these positions as that of H7N9_ZJ13_, suggesting that these sites were not responsible for eqMx1 restriction (Figure 5A). The other six residues were selected for single point mutations in both H3N8_JL89_ NP and H7N9_ZJ13_ NP. In H3N8_JL89_ NP, the mutants were G34S, G50S, H52N, K77R, M105V, and V186I. We found mutations G34S (albeit not statistically significant) and H52N altered the restriction of eqMx1, whereas the mutants G50S, K77R, M105V, and V186I remained completely unaffected by eqMx1. The effect of these point mutations was also confirmed by creating reverse mutations in the H7N9_ZJ13_ NP. As expected, the reverse effect was observed for the mutation at site 52 of H7N9_ZJ13_ NP (N52H) (Figure 5B). However, interestingly, mutating S34G in the H7N9_ZJ13_ NP had no distinguishable alteration in the activity towards eqMx1, rather a markedly less resistant phenotype was observed and the mutant behaved like a wild type H7N9_ZJ13_ NP. Similarly, mutations at S50G, R77K, V105M, and I186V of H7N9_ZJ13_ NP showed no effect and mutation I186V showed more inhibition towards eqMx1. These results indicate that position 52 of equine IAV is important for NP adaptation to eqMx1.

In order to confirm the importance of site 52, common to both H3N8_JL89_ NP and H7N9_ZJ13_ NP, the specific mutations were introduced into H5N1 NP and tested for its influence on eqMx1 resistance in the H5N1 polymerase reconstitution assay. H5N1 was chosen because it is another avian IAV that is blocked by eqMx1. H5N1 contains Tyrosine (Y) at position 52 (Figure 5C). As expected, point mutations in H3N8_JL89_ NP (H52Y) and H5N1 NP (Y52H) showed a reverse effect against eqMx1, confirming the importance of position 52. In order to determine the effect of the two identified sites 34 and 52 in H3N8_JL89_ in resistance against huMxA, the polymerase activity of three H3N8_JL89_ NP mutants G34S, H52N, and H52Y, as well as two H7N9_ZJ13_ NP mutants S34G and N52H, were measured. The mutants G34S, H52N, and H52Y showed less pronounced polymerase activity than the wild type H3N8_JL89_ NP but still could not escape the antiviral activity of huMxA (Figure 5D). Similarly, reverse mutations in H7N9_ZJ13_ NP, S34G, and N52H did not alter the sensitivity to huMxA (Figure 5E). A decent expression level of all the NP mutant plasmids was detected using western blotting. Together, these results suggested that the amino acids at position 52 of NPs from different IAVs conferred Mx1 resistance.

### 3.4. The Amino Acid Position 52 of NP Affects its Interaction with eqMx1

**Figure 6:**
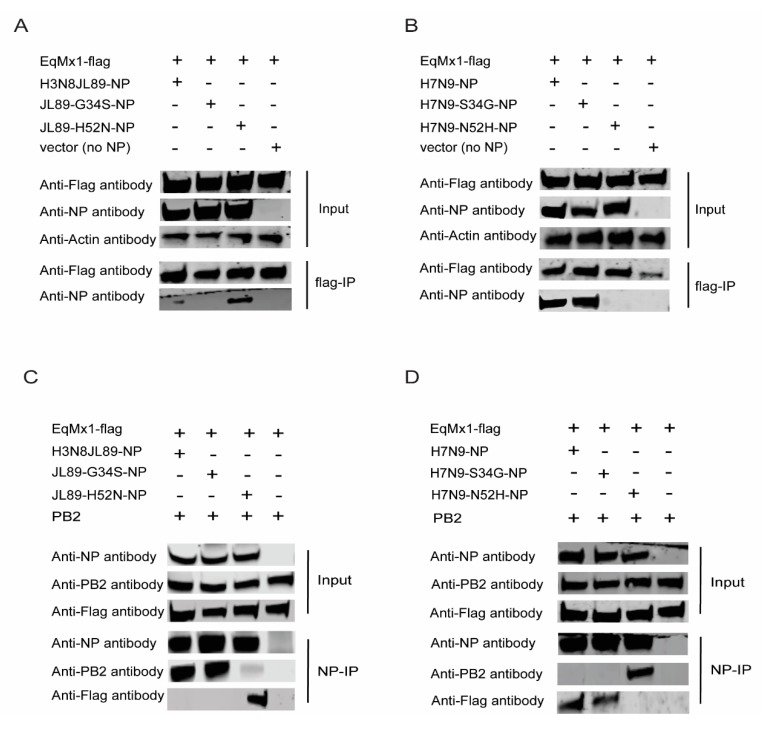
Co-immunoprecipitation of eqMx1 and NP/PB2. We carried out this experiment by doing a pull-down of the NP followed by the analysis of co-immunoprecipitated PB2 using HEK293T cells that were co-transfected with p3XFLAG- eqMx1 (4 μg) together with PB1, PB2 (2 μg each), PA, FF-Luc (1 μg each), and NP expression plasmids from H7N9_ZJ13_ or its mutants H7N9 _ZJ13_-S34G, N52H; or H3N8_JL89_ or its mutants H3N8_JL89_-G34S, H52N; or an empty control vector (4 μg each). Total lysates were made 24 hr after transfection, HEK293T cells lysates were assayed by FLAG-IP (**A**,**B**) or NP-IP (**C**,**D**) and blotted with the indicated antibodies. The PB2 antibody was acquired from NOVUS Biologicals, Centennial, Colorado, USA (NBP2-42879). For western blot analysis, actin was used as a loading control. All experiments were repeated three times and representative figures are shown.

To further confirm the interaction between NP and eqMx1 and the importance of site 52 of the NP in determining this interaction, we conducted a co-immunoprecipitation (Co-IP) experiment using different NP proteins and eqMx1 protein. Flag-tagged eqMx1 was co-expressed in HEK293T cells, individually or in combination with expression plasmids of PB1, PB2, PA, viral reporter RNA (FF-luc), and NP from either H3N8_JL89_ and mutants (H3N8_JL89_-G34S-NP, H3N8_JL89_-H52N-NP) or H7N9_ZJ13_ and its mutants (H7N9_ZJ13_-S34G-NP, H7N9_ZJ13_-N52H-NP), to mimic the viral vRNPs. Twenty four hours post-transfection, total cell lysates were harvested from transfected cells, and western blotting was performed after FLAG-IP using Anti-FLAG M2 magnetic beads (Sigma Aldrich, St. Louis, MO, USA, M8823). Results showed that when the eqMx1 was co-expressed with either H3N8_JL89_-NP or H3N8_JL89_-G34S-NP, no interaction was identified. Expectedly, the mutant H3N8_JL89_-H52N-NP showed a strong interaction with eqMx1 (Figure 6A). In contrast, Flag-tagged eqMx1 was co-immunoprecipitated with NP from H7N9_ZJ13_ and its mutant –S34G when they were co-expressed. This interaction was lost when site 52 mutated in H7N9_ZJ13_ to–N52H (Figure 6B). This finding gave evidence to the hypothesis that position 52 could be the most important site for interaction between eqMx1 and NP of IAV.

To confirm this result, the reverse Co-IP experiment with Anti-NP magnetic beads (MCE protein A/G magnetic beads and NP antibody from our lab) was done (Figure 6C,D) and the results confirmed the results obtained in Figure 6A,B. It has been previously reported that interaction between PB2 and NP is crucial for the establishment of an enzymatically-active influenza RdRp in RNP complexes [42], and that enzymatically-active Mx1 blocks viral replication by blocking the PB2-NP interface, signifying its direct effect on the vRNP complex. Therefore, we analyzed the PB2-NP interaction in the IP complexes in the presence of eqMx1. No PB2 was found co-immunoprecipitated with H3N8_JL89_-H52N-NP, H7N9_ZJ13_-NP, and H7N9_ZJ13_-S34G. On the other hand, in the complex where there was no interaction between eqMx1 and NP (H3N8_JL89_,-G34S and H7N9_ZJ13_,-N52H) PB2 was co-immunoprecipitated with the said variants of NP (Figure 6C,D; lanes 4 to 6). This result supported the model in which eqMx1 interacts with the influenza RNP and affects its assembly by disturbing the PB2-NP interaction [6]. Together these results reveal a fundamental function of site 52 in altering IAV polymerase activity in the presence of eqMx1, as well as its interaction with eqMx1.

### 3.5. A Single Mutation at Position 52 in H3N8_JL89_ NP Allows the Virus to escape from eqMx1 Restriction

**Figure 7:**
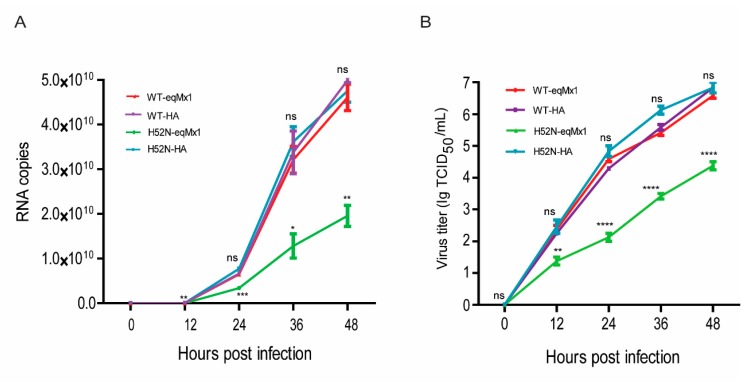
The replication abilities of viruses with different NPs underexpression of eqMx1. (**A**) MDCK cells expressing HA-tagged eqMx1 were infected with wild type (H3N8_JL89_) or mutant viruses (H3N8_JL89-_H52N-NP) at an MOI of 0.001 and the supernatants were collected at 0, 12, 24, 36, and 48 hr post-infection. Total RNA from the collected supernatants was extracted using the RNeasy plus mini kit (Qiagen) and subjected to one-step real-time quantitative PCR (qPCR) analysis using the AgPath-ID™ One-Step RT-PCR reagents according to manufacturer’s protocol. Relative mRNA expression levels were determined using double-standard curve methods. All the experiments were performed three times and with three replicates with means ± SE shown. (**B**) MDCK cells expressing eqMx1 were infected with wild type (H3N8_JL89_) or mutant viruses (H3N8_JL89_-H52N-NP) at an MOI of 0.001 and the supernatants were collected at 0, 12, 24, 36, and 48 hr post-infection. These supernatants were subsequently used to infect MDCK cells at different dilutions (10^−1^ to 10^−11^) with at least four repeats. 48 hr post-infection, immunofluorescence assays (IFA) were performed using specific antibodies against viral NP (from our lab) and FITC-labelled secondary antibody. Finally the viral titers were calculated using Reed and Munech methodology and results are shown as TCID_50_/mL. The results of a single experiment performed with four repeats are shown and results were subsequently confirmed in three separate experiments. Error bars indicate standard deviations (SD).

To further evaluate the importance of 52H of the NP protein in the resistance of eqMx1, we rescued viruses H3N8_JL89_ and H3N8_JL89_ with an H52N substitution in the NP using a reverse genetic system as previously described [34]. Viral titers were calculated by means of the Reed and Muench methodology [36]. MDCK cells expressing HA-tagged pcDNA3.1-eqMx1 or HA-tagged pcDNA3.1 empty control vector, were infected at an MOI of 0.001 for a duration of 1 hr with these harvested viruses and the supernatants were collected at 0, 12, 24, 36, and 48 hr post-infection, and RNA was extracted. Real-time quantitative PCR (qPCR) was performed to determine relative mRNA expression levels. We found that the wild type H3N8_JL89_ had almost the same number of copies in the presence or absence of eqMx1 during replication, whereas the number of copies of mutant virus (JL89-H52N) were drastically reduced in the cells expressing eqMx1, indicating that this mutant virus strain could be blocked by eqMx1, in contrast to the wild type H3N8_JL89_ virus (Figure 7A). Additionally, viral titers were measured using TCID_50_ /mL and the results were in accordance with the qPCR analysis (Figure 7B). From these data we can conclude that a single position in the NP of the H3N8_JL89_ virus (position 52) is essential for viral replication and to allow the virus to escape the restriction effect of eqMx1.

## 4. Discussion

The interferon-induced huMxA protein signifies a key interspecies barrier for a large number of zoonotic viruses, including IAVs [8]. Equine Mx1, encoded by a gene located on chromosome 26, is a homolog of MxA from humans or other hosts and has been poorly studied [43,44]. A recent study on the comparison of antiviral activity of Mx1 from different host species includes the corresponding gene from equines and suggests that its antiviral activity against the influenza virus was similar to Mx1 from water buffalo [14]. Here we firstly compared the antiviral potentials of Mx1 proteins from human and equine hosts and identified the potential eqMx1 to inhibit IAV from different species in a species-specific manner. We further identified that NP is responsible for interaction with eqMx1 and confers resistance to the restriction of eqMx1. A single amino acid mutation H52N of NP alters this resistance and interaction to eqMx1 and changed the viral replication ability when eqMx1 was present.

Equine H3N8_JL89_ is predicted to be evolved from an avian origin and equine IAV epizootics has a relation to IAV epidemics in humans and canines [45]. The equine IAV H3N8 strain has been seen to affect dog populations, indicating its efficiency in switching stable hosts and to evolve under antigenic drift [46]. The H3N8 virus has also been isolated from a swine population in China, but this has not been confirmed as a stable host switch, and sequence and phylogenetic analyses of eight gene segments showed that the two swine isolates were of equine origin and most closely related to European equine H3N8 influenza viruses from the early 1990s [47]. All this evidence is of rare cases and the equine IAVs keep an independent evolutionary lineage compared with IAVs from other species.

NP is known as a key module of the vRNP complex and is indispensable for virus replication. NP is the most conserved protein within IAV viral proteins and was identified as a determinant of viral sensitivity towards Mx1 proteins in previously conducted studies, for example, site mutations at 48Q, 98K, and 99K in A/swine/Belzig/2/2001 are sufficient to provide resistance to huMxA [20,21,22]. By using polymerase reconstitution assays, we found two positions in H3N8_JL89_ NP, G34, and H52, which could be a possible target for H3N8_JL89_ IAV. In our study, we showed that in the H7N9_ZJ13_ NP the mutation N52H enabled it to escape the eqMx1 restriction. Interestingly, the S34G substitution in the H7N9_ZJ13_ did not reverse the activity against eqMx1. Substituting tyrosine (Y) at position 52 with histidine (H) in H5N1 altered the polymerase activity of H5N1 drastically and eqMx1 almost lost its restriction affect towards H5N1-Y52H. A similar substitution in H3N8_JL89_ NP (H3N8_JL89_-H52Y) produced a reverse effect and eqMx1 inhibited the polymerase activity, confirming the importance of this site. We concluded that site 52 is more universal for eqMx1 as it was found to be of equal importance in both IAV strains, whereas site 34 was found to alter the polymerase activity only of H3N8_JL89_ against eqMx1. The sequence alignment of unique sequences of H7N9 and H3N8 predicted that position 52 was highly conserved in both strains, whereas site 34 was more prone to mutations in both strains, reiterating the importance of site 52. None of the mutations at sites 34 and 52 in either H3N8_JL89_ or H7N9_ZJ13_ had a significant effect against huMxA but showed a slight alternation of the restriction instead. These sites may be important in escaping the huMxA antiviral activity when in combination with other as yet unidentified sites.

The NP and PB2 have been found to have a major role in blocking the antiviral effect of murine Mx1 [18,19,20]. As previously reported, the interaction between PB2 and PB1 is not influenced by the presence of the Mx1 protein. Mx1 inhibits the interaction between PB2 and NP, probably leaving the ternary RdRp complex intact, suggesting that Mx1 inhibits the interaction between the RdRp and the NP protein in vRNP complexes [6,18]. Our results helped to explain how eqMx1 could block viral replication by interacting with viral NPs, thus blocking NP-PB2 interaction and explaining how a single mutation could affect the binding of two proteins.

The interaction of Mx1 with PB2 and NP could be direct or indirect. Other cellular proteins might be involved in this interaction and could act as a bridge between Mx1 and the vRNP complex. Only a few proteins have been reported to interact with the Mx1 protein, and these interacting proteins are potential candidates as bridging factors [48]. Multiple cellular proteins interact with PB2 and/or NP and might fulfill this function. The aforementioned experiments clearly demonstrate an interaction between Mx1 and PB2 or NP when all components of the minireplicon system are present, but Verhelst, Judith, et al. have reported that this interaction between Mx1 and PB2 or NP could occur in the presence or absence of other viral proteins. The authors also reported that interactions between Mx1 and PB2 or NP on the other cannot be detected in the absence of NEM [6]. We also observed a similar result during the IP experiment (data not shown). NEM might preserve the conformation of viral proteins or a cellular factor that mediates the interaction between Mx1 and PB2 or NP. It is also possible that the interactions are too transient or too weak, and inhibiting the GTPase activity of Mx1 by using NEM could stabilize these interactions.

The HEK293T cell line was selected as the first choice for the transfection of plasmids throughout the study except for virus infection experiments because the HEK293T cells are well used in previous studies [6,38,49,50]. Since influenza viruses are the pathogens that cause major respiratory tract infections, the lung epithelial cells might be used for in vitro analysis of host-virus interaction at the cellular level. We tried to transfect human lung epithelial A549 cells but found the transfection efficiency was very low (only about 10–20%). Additionally, when compared with the endogenous MxA in both cell lines using MX1 rabbit polyclonal antibody (Protientech, Rosemont, Illinois, USA), A549 cells did express notable levels of endogenous human MxA that might interfere with our results (see Figure S1). A validation of interaction between eqMx1 and viral NP, and the site-specific antagonism of different viral strains, in lung epithelial cells of equine origin might be a future perspective in order to clarify the mode of action of equine Mx1 in vitro.

To summarize, like its other homologs, eqMx1 could be an efficient barrier against the transmission of IAVs into the equine population. The anti-influenza activities of eqMx1 were species-specific and the NP was the major target of eqMx1. The key sites on H3N8_JL89_ NP determined the sensitivity and resistance to eqMx1. Further extensive studies are required to demonstrate the exact molecular mechanism involved in the structure-specific interaction and evolution, which may deliver new aims for antiviral intervention.

## Supplementary Materials

The following are available online at https://www.mdpi.com/1999-4915/11/12/1114/s1, Table S1: Equine Mx1 primers, Table S2: Chimeric clones primers, Table S3: Point Mutation primers, Figure S1 western blot image of huMx1 endogenous expression level in A549 and 293T cells.

## References

[1] Taubenberger J.K., Kash J.C. (2010). Influenza virus evolution, host adaptation, and pandemic formation. Cell Host Microbe.

[2] Horimoto T., Kawaoka Y. (2001). Pandemic threat posed by avian influenza A viruses. Clin. Microbiol. Rev..

[3] Short K.R., Richard M., Verhagen J.H., van Riel D., Schrauwen E.J., van den Brand J.M., Mänz B., Bodewes R., Herfst S. (2015). One health, multiple challenges: The inter-species transmission of influenza A virus. One Health.

[4] Webster R.G., Bean W.J., Gorman O.T., Chambers T.M., Kawaoka Y. (1992). Evolution and ecology of influenza A viruses. Microbiol. Mol. Biol. Rev..

[5] Mostafa A., Abdelwhab E.M., Mettenleiter T.C., Pleschka S. (2018). Zoonotic potential of influenza A viruses: A comprehensive overview. Viruses.

[6] Verhelst J., Parthoens E., Schepens B., Fiers W., Saelens X. (2012). Interferon-inducible protein Mx1 inhibits influenza virus by interfering with functional viral ribonucleoprotein complex assembly. J. Virol..

[7] Haller O., Staeheli P., Schwemmle M., Kochs G. (2015). Mx GTPases: Dynamin-like antiviral machines of innate immunity. Trends Microbiol..

[8] Verhelst J., Hulpiau P., Saelens X. (2013). Mx proteins: Antiviral gatekeepers that restrain the uninvited. Microbiol. Mol. Biol. Rev..

[9] Xiao H., Killip M.J., Staeheli P., Randall R.E., Jackson D. (2013). The human interferon-induced MxA protein inhibits early stages of influenza A virus infection by retaining the incoming viral genome in the cytoplasm. J. Virol..

[10] Pavlovic J., Haller O., Staeheli P. (1992). Human and mouse Mx proteins inhibit different steps of the influenza virus multiplication cycle. J. Virol..

[11] Schneider-Schaulies S., Schneider-Schaulies J., Schuster A., Bayer M., Pavlovic J., Ter Meulen V. (1994). Cell type-specific MxA-mediated inhibition of measles virus transcription in human brain cells. J. Virol..

[12] Kochs G., Janzen C., Hohenberg H., Haller O. (2002). Antivirally active MxA protein sequesters La Crosse virus nucleocapsid protein into perinuclear complexes. Proc. Natl. Acad. Sci. USA.

[13] Frese M., Kochs G., Feldmann H., Hertkorn C., Haller O. (1996). Inhibition of bunyaviruses, phleboviruses, and hantaviruses by human MxA protein. J. Virol..

[14] Dam Van P., Desmecht D., Garigliany M.-M., Bui Tran Anh D., Van Laere A.-S. (2019). Anti-Influenza A Virus Activities of Type I/III Interferons-Induced Mx1 GTPases from Different Mammalian Species. J. Interferon Cytokine Res..

[15] Gao S., von der Malsburg A., Dick A., Faelber K., Schröder G.F., Haller O., Kochs G., Daumke O. (2011). Structure of myxovirus resistance protein a reveals intra-and intermolecular domain interactions required for the antiviral function. Immunity.

[16] Gao S., von der Malsburg A., Paeschke S., Behlke J., Haller O., Kochs G., Daumke O. (2010). Structural basis of oligomerization in the stalk region of dynamin-like MxA. Nature.

[17] Daumke O., Gao S., von der Malsburg A., Haller O., Kochs G. (2010). Structure of the MxA stalk elucidates the assembly of ring-like units of an antiviral module. Small GTPases.

[18] Stranden A.M., Staeheli P., Pavlovic J. (1993). Function of the mouse Mx1 protein is inhibited by overexpression of the PB2 protein of influenza virus. Virology.

[19] Huang T., Pavlovic J., Staeheli P., Krystal M. (1992). Overexpression of the influenza virus polymerase can titrate out inhibition by the murine Mx1 protein. J. Virol..

[20] Mänz B., Dornfeld D., Götz V., Zell R., Zimmermann P., Haller O., Kochs G., Schwemmle M. (2013). Pandemic influenza A viruses escape from restriction by human MxA through adaptive mutations in the nucleoprotein. PLoS Pathog..

[21] Dittmann J., Stertz S., Grimm D., Steel J., García-Sastre A., Haller O., Kochs G. (2008). Influenza A virus strains differ in sensitivity to the antiviral action of Mx-GTPase. J. Virol..

[22] Dornfeld D., Petric P.P., Hassan E., Zell R., Schwemmle M. (2019). Eurasian Avian-Like Swine Influenza A Viruses Escape Human MxA Restriction through Distinct Mutations in Their Nucleoprotein. J. Virol..

[23] Asano A., Ko J.H., Morozumi T., Hamashima N., Watanabe T. (2002). Polymorphisms and the antiviral property of porcine Mx1 protein. J. Vet. Med. Sci..

[24] Babiker H., Nakatsu Y., Yamada K., Yoneda A., Takada A., Ueda J., Hata H., Watanabe T. (2007). Bovine and water buffalo Mx2 genes: Polymorphism and antiviral activity. Immunogenetics.

[25] Baise E., Pire G., Leroy M., Gérardin J., Goris N., Clercq K.D., Kerkhofs P., Desmecht D. (2004). Conditional expression of type I interferon-induced bovine Mx1 GTPase in a stable transgenic vero cell line interferes with replication of vesicular stomatitis virus. J. Interferon Cytokine Res..

[26] Yamada K., Nakatsu Y., Onogi A., Ueda J., Watanabe T. (2009). Specific intracellular localization and antiviral property of genetic and splicing variants in bovine Mx1. Viral Immunol..

[27] Riegger D., Hai R., Dornfeld D., Mänz B., Leyva-Grado V., Sánchez-Aparicio M.T., Albrecht R.A., Palese P., Haller O., Schwemmle M. (2015). The nucleoprotein of newly emerged H7N9 influenza A virus harbors a unique motif conferring resistance to antiviral human MxA. J. Virol..

[28] Götz V., Magar L., Dornfeld D., Giese S., Pohlmann A., Höper D., Kong B.-W., Jans D.A., Beer M., Haller O. (2016). Influenza A viruses escape from MxA restriction at the expense of efficient nuclear vRNP import. Sci. Rep..

[29] Deeg C.M., Hassan E., Mutz P., Rheinemann L., Götz V., Magar L., Schilling M., Kallfass C., Nürnberger C., Soubies S. (2017). In vivo evasion of MxA by avian influenza viruses requires human signature in the viral nucleoprotein. J. Exp. Med..

[30] Mino S., Mojsiejczuk L., Guo W., Zhang H., Qi T., Du C., Zhang X., Wang J., Campos R., Wang X. (2019). Equine Influenza Virus in Asia: Phylogeographic Pattern and Molecular Features Reveal Circulation of an Autochthonous Lineage. J. Virol..

[31] Sovinova O., Tumova B., Pouska F., Nemec J. (1958). Isolation of a virus causing respiratory disease in horses. Acta Virol..

[32] Gorman O., Bean W., Kawaoka Y., Donatelli I., Guo Y., Webster R. (1991). Evolution of influenza A virus nucleoprotein genes: Implications for the origins of H1N1 human and classical swine viruses. J. Virol..

[33] Lin Y.-Z., Cao X.-Z., Li L., Li L., Jiang C.-G., Wang X.-F., Ma J., Zhou J.-H. (2011). The pathogenic and vaccine strains of equine infectious anemia virus differentially induce cytokine and chemokine expression and apoptosis in macrophages. Virus Res..

[34] Zimmermann P., Mänz B., Haller O., Schwemmle M., Kochs G. (2011). The viral nucleoprotein determines Mx sensitivity of influenza A viruses. J. Virol..

[35] Pleschka S., Jaskunas R., Engelhardt O.G., Zürcher T., Palese P., Garcia-Sastre A. (1996). A plasmid-based reverse genetics system for influenza A virus. J. Virol..

[36] Reed L.J., Muench H. (1938). A simple method of estimating fifty per cent endpoints12. Am. J. Epidemiol..

[37] Zhang H., Zhang Z., Wang Y., Wang M., Wang X., Zhang X., Ji S., Du C., Chen H., Wang X. (2019). Fundamental contribution and host range determination of ANP32 protein family in influenza A virus polymerase activity. bioRxiv.

[38] Verhelst J., De Vlieger D., Saelens X. (2015). Co-immunoprecipitation of the mouse Mx1 protein with the influenza a virus nucleoprotein. JoVE (J. Vis. Exp.).

[39] Wang M., Zhang Z., Wang X. (2018). Strain-specific antagonism of the human H1N1 influenza A virus against equine tetherin. Viruses.

[40] Kumar S., Tamura K., Nei M. (2004). MEGA3: Integrated software for molecular evolutionary genetics analysis and sequence alignment. Brief. Bioinform..

[41] Ashenberg O., Padmakumar J., Doud M.B., Bloom J.D. (2017). Deep mutational scanning identifies sites in influenza nucleoprotein that affect viral inhibition by MxA. PLoS Pathog..

[42] Biswas S.K., Boutz P.L., Nayak D.P. (1998). Influenza virus nucleoprotein interacts with influenza virus polymerase proteins. J. Virol..

[43] Lear T., Breen M., Ponce de Leon F., Coogle L., Ferguson E., Chambers T., Bailey E. (1998). Cloning and chromosomal localization of MX1 and ETS2 to chromosome 26 of the horse (Equus caballus). Chromosome Res..

[44] Heinz H., Marquardt J., Schuberth H.-J., Adolf G., Leibold W. (1994). Proteins induced by recombinant equine interferon-β1 within equine peripheral blood mononuclear cells and polymorphonuclear neutrophilic granulocytes. Vet. Immunol. Immunopathol..

[45] Taubenberger J., Morens D. (2009). Pandemic influenza–including a risk assessment of H5N1. Rev. Sci. Tech. (Int. Off. Epizoot.).

[46] Crawford P., Dubovi E.J., Castleman W.L., Stephenson I., Gibbs E., Chen L., Smith C., Hill R.C., Ferro P., Pompey J. (2005). Transmission of equine influenza virus to dogs. Science.

[47] Tu J., Zhou H., Jiang T., Li C., Zhang A., Guo X., Zou W., Chen H., Jin M. (2009). Isolation and molecular characterization of equine H3N8 influenza viruses from pigs in China. Arch. Virol..

[48] Engelhardt O.G., Ullrich E., Kochs G., Haller O. (2001). Interferon-induced antiviral Mx1 GTPase is associated with components of the SUMO-1 system and promyelocytic leukemia protein nuclear bodies. Exp. Cell Res..

[49] Patzina C., Haller O., Kochs G. (2014). Structural requirements for the antiviral activity of the human MxA protein against Thogoto and influenza A virus. J. Biol. Chem..

[50] Giese S., Ciminski K., Bolte H., Moreira É.A., Lakdawala S., Hu Z., David Q., Kolesnikova L., Götz V., Zhao Y. (2017). Role of influenza A virus NP acetylation on viral growth and replication. Nat. Commun..

