# Supplementary material for: Equine Mx1 Restricts Influenza A Virus Replication by Targeting at Distinct Site of its Nucleoprotein
Source: Viruses. 2019 Dec 2;11(12):1114. doi: 10.3390/v11121114 (PMC6950424; doi:10.3390/v11121114)
Supplement: Supplementary file 1 [file viruses-11-01114-s001.zip › viruses-614159-suppl/Figure S1.pdf]

Fig S1

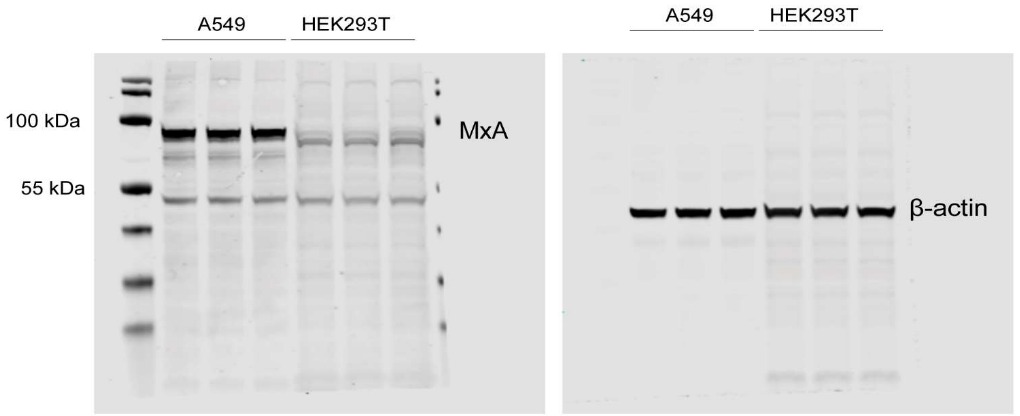

Figure S1: (A) Levels of endogenous human MxA in A549 and HEK293T cells, (B) levels of β-actin, same samples were loaded on different gel to get clear bands of internal control
