# Supplementary material for: Equine Mx1 Restricts Influenza A Virus Replication by Targeting at Distinct Site of its Nucleoprotein
Source: Viruses. 2019 Dec 2;11(12):1114. doi: 10.3390/v11121114 (PMC6950424; doi:10.3390/v11121114)
Supplement: Supplementary file 1 [file viruses-11-01114-s001.zip › viruses-614159-suppl/suppliermentry tables.pdf]

TABLE S1:

PRIMERS FOR EQUINE MX1

NAME	SEQUENCE
EqMx1-F	ATGGTTCATTCTGAAGCGAAAATGACAAGA
EqMx1-R	TTAACCCGGGAACTTGGCTAACCGGCGCCG

TABLE S2:

CHIMERIC CLONES PRIMERS

NAME	SEQUENCE	TEMPLATE USED
N-H7N9xJL-CT vet down	atggcgtctcaaggcaccaaacgatcttatgaacagatg	H7N9-NP
N-H7N9xJL-CT vet up	cccccgctttgtcatccgaattagttccatcaccattgt	H7N9-NP
N-H7N9xJL-CT int F	aagcgggggatcaatgatcgaaatttctggaga	JL89-NP
N-H7N9xJL-CT int R	attgtcatattcctctgcattgtctccgaagaa	JL89-NP
N-JL89xH7N9-C vet up	atggcgtctcaaggcaccaaacgatcctatgaa	JL89-NP
N-JL89xH7N9-C) vet down	ccctcgttttatcatccgaatcagttccatcac	JL89-NP
N-JL89xH7N9-C int f	aaacgagggatcaacgaccggaatttctggaga	H7N9-NP
N-JL89xH7N9-C int R	tcaattgtcatactcctctgcattgtctccgaa	H7N9-NP

TABLE S3:

SINGLE MUTATIONS

NAME	SEQUENCE
JL89-G34S F	gttggaagaatggttAgtggaattgggaggttctacat
JL89-G34S R	atgtagaacctcccaattccacTaaccattcttccaac
JL89-G50S F	gcactgaactcaagctcAgcgaccatgaagggaggctg
JL89-G50S F	cagcctcccttcatggtcgcTgagcttgagttcagtgc
JL89-H52Y F	AGCTCGGCGACtATGAAGGGAGGCTGATTCAG
JL89-H52Y R	TCCCTTCATaGTCGCCGAGCTTGAGTTCAG
JL89-H52N F	GCTCGGCGACaATGAAGGGAGGCTGATTCA
JL89-H52N R	TCCCTTCATtGTCGCCGAGCTTGAGTTCAG
JL89-K77R F	tttgatgaaaggaggaacaGatacctggaagaacatc
JL89-K77R R	gatgttcttccaggtatCtgttcctcctttcatcaaa
JL89-M105V F	agagatgggaaatggGtgagagagctgattctg
JL89-M105V R	cagaatcagctctctcaCccatttcccatctct
JL89-V186I F	gcagcagtaaagggaAtcggaacaatggtgatg
JL89-V186I R	catcaccattgttccgaTtccctttactgctgc
**NAME**	**SEQUENCE**
H7N9-S34G F	agaatggttGgcggcattgggagattctac
H7N9-S34G R	gtagaatctcccaatgccgcCaaccattct
H7N9-S50G F	tgtacagaactcaaactcGgtgac
H7N9-S50G R	gtcacCgagtttgagttctgt
H7N9-N52H F	gacCatgaagggaggctgatt
H7N9-N52H R	aatcagcctcccttcatGgtc
H7N9-R77K F	tttgatgaaagaaggaacaAatacctggaagagcac
H7N9-R77K R	gtgctcttccaggtatTtgttccttctttcatcaaa
H7N9-V105M F	agagacggaaaatggAtgagagagctgatcctgt

H7N9-V105M R	acaggatcagctctctcaTccattttccgtctct
H7N9-I186V F	gcagcagtgaaggggGtagggacaatggtg
H7N9-I186V R	caccattgtccctaCcccttcactgctgc
H5N1-Y52H F	gaactcaaactcagtgacCatgaagggagactg
H5N1-Y52H R	cagtctcccttcatGgtcactgagtttgagttc
